# Silymarin and Fatty Acid Profiles of Milk Thistle (*Silybum marianum* L.) Genotypes

**DOI:** 10.1007/s11130-025-01400-0

**Published:** 2025-09-15

**Authors:** Barbora Kudláčková, Petr Misák, Helena Pluháčková

**Affiliations:** 1 Institute of Analytical Chemistry of the Czech Academy of Sciences, Veveří 967/97, Brno, 602 00 Czech Republic; 2 Department of Crop Science, Breeding and Plant Medicine, Mendel University in Brno, Zemědělská 1665/1, Brno, 613 00 Czech Republic; 3 Faculty of Civil Engineering, Brno University of Technology, Veveří 331/95, Brno, 602 00 Czech Republic

**Keywords:** *Silybum marianum* L., Silymarin complex, Oil, Fatty acids

## Abstract

Milk thistle (*Silybum marianum* L.) seeds are known for their high amounts of bioactive silymarin complex and oil, both contributing to its potential as a functional food. This study aimed to evaluate the phytochemical composition of four milk thistle varieties (Silma, Silyb, Mirel, and Moravia 55), focusing on the silymarin complex, oil yield, and fatty acid profile. Silymarin content was quantified by high-performance liquid chromatography with diode-array detection (HPLC-DAD) following ultrasound-assisted extraction (USE). Pressurized liquid extraction (PLE) was employed for determining oil yield, and the fatty acid composition was performed by gas chromatography with flame ionization detection (GC-FID). Results showed significant variability between genotypes. The total silymarin content ranged from 12.69 to 20.28 mg·g⁻¹ DW, with silychristin, silybin A, and silybin B as major constituents. Oil content varied from 22.81 to 26.25%, with Mirel 2 showing the highest yield. Fatty acid analysis revealed a high proportion of unsaturated fatty acids (79.30–83.30%), with linoleic (53.53–62.27%) and oleic (20.17–24.57%) acids being the most abundant. The results were further evaluated by Principal Component Analysis and hierarchical cluster analysis. The study’s findings may contribute to the strategic selection of milk thistle genotypes for pharmaceutical or nutritional applications, and support targeted cultivation to optimize phytochemical content.

**Supplementary Information:**

The online version contains supplementary material available at 10.1007/s11130-025-01400-0.

## Introduction

*Silybum marianum* L. (known also as milk thistle), a member of the *Asteraceae* family, has been used for centuries in traditional medicine to treat a variety of diseases. It is widely used for its hepatoprotective, antioxidant, and anti-inflammatory properties, which are primarily attributed to the presence of silymarin complex - a unique group of flavonolignans including silychristin, silydianin, silybin A, B, isosilybin A, B, concentrated in the achenes of the plant. Additionally, milk thistle achenes contain flavonoids such as quercetin and taxifolin, as well as phenolic acids like chlorogenic acid and caffeic acid [1, 2]. These compounds collectively exhibit potent free radical-scavenging activity and membrane-stabilizing effects, making milk thistle a valuable therapeutic agent for managing liver diseases, including hepatitis, cirrhosis, and toxin-induced liver damage [3, 4]. In addition to its high silymarin complex content, milk thistle achenes are also known as a rich source of oil, comprising approximately 20–30% of their weight, with linoleic (C18:2) and oleic (C18:1) as the main unsaturated fatty acids [2, 5]. The regular intake of these two fatty acids is associated with a decreased risk of cardiovascular diseases, as the most common age-related diseases in the world [6, 7]. The milk thistle achenes are also a valuable source of proteins, fiber, and minerals, further enhancing their nutritional value. These components not only contribute to the plant’s pharmacological effects but also expand its applications beyond medicine, making it a versatile raw material for the nutraceutical, cosmetic, and food industries [5]. Furthermore, the oil from milk thistle seeds is gaining attention for its potential use in biofuels and other bio-based products, adding to its economic and environmental significance [8, 9]. Therefore, determination of the silymarin complex, oil content, and fatty acid composition is crucial for assessing the quality and therapeutic potential of milk thistle seeds. These parameters not only provide insights into the biochemical profile of the plant but also serve as quality markers for raw materials and finished products derived from milk thistle.

Milk thistle is the dominant medicinal crop in the Czech Republic, where it is cultivated on approximately 2,000 ha with an average yield of 0.40 t/ha [10]. A key feature of its production is the emphasis on quality control. Therefore, the cultivation is performed to order, and processors directly supply seed material to ensure a uniform phytochemical composition suitable for specific end-products (e.g., oil, standardized extracts). This industrial focus is paralleled by ongoing plant breeding processes that have resulted now in ten nationally protected varieties (Mirel, Moravia 55, Aida, Tevasil, Albus, Tevadian, Dominicus, Michael, Mirel plus, and Silygreen). Mirel variety, owned by Moravol, spol. s.r.o., CZ, is valued primarily for its high oil content and specific fatty acid composition. The Moravia 55, Mirel plus, and Dominicus varieties are also owned by Moravol, spol. s.r.o., CZ, which cultivates and processes it for its own purposes. To the best of our knowledge, this variety has not been extensively characterized in the scientific literature. The Michael, Syligreen, and Aida varieties, owned by Irel, spol. s.r.o., are cultivated for their own pharmaceutical and nutritional processing. Information about their phytochemical composition is also unknown. The Aida, Tevasil, and Tevadian genotypes are co—owned by TAPI Czech Industries s.r.o. and Palacký University Olomouc. Both the Tevasil and Albus varieties are known for their high silymarin content, with Albus also being phenotypically distinct due to its rapid fruition and white flowers, distinguishing it from traditional purple varieties. The Tevadian variety is characterized by a high silydianin content, to the detriment of silybin [11, 12]. The composition, nutritional, and functional properties of Tevasil and Tevadian cultivars were recently described by Bártová et al. [11]. Evaluation of the chemical profile of individual varieties is then essential not only for product development but also for agricultural practices. Modern approaches of milk thistle cultivation increasingly emphasize sustainability, precise fertilization, and adaptive responses to climate changes with ensuring consistent yields and high-quality raw material. This approach requires close collaboration between researchers and the agricultural community.

Based on these, this study aimed to determine the phytochemical composition (silymarin content, oil yield, and fatty acid profile) of four milk thistle varieties, Silma, Silyb, Mirel, and Moravia 55, to evaluate their potential for pharmaceutical, dietary applications, or future breeding research.

## Materials and Methods

The material and methods section is described in Supplementary Material S1.

### Plant Material

Milk thistle achenes of the varieties Silma, Silyb, Mirel, and Moravia 55 at the whole stage of maturity were obtained from various growers in the Czech Republic or Slovakia. The moisture content in the seeds was approximately 5% and TSW varied in the range from 23.0 to 28.6 g (Table S1). Precise information about the field location, soil, or fertilization of individual milk thistle samples are unknown. Prior to analysis, all the achenes were ground for 30 s using an IKA Tube Mill 100 control (IKA^®^-Werke GmbH & Co. KG, Germany) at 20, 000 rpm, the particle size of the achenes was ≤ 0.5 mm.

## Results and Discussion

### Silymarin Complex Profile of Milk Thistle Achenes

The silymarin complex profile of the individual milk thistle achenes represents an important characterization of their nutritional and pharmacological properties. According to the obtained results (Table 1), statistically significant differences were observed in the silymarin complex content depending on the variety (*p* ˂ 0.001). The total silymarin complex content across the varieties ranged from 12.69 mg.g^−1^ DW (Moravia 55) to 20.28 mg.g^−1^ DW (Silma). In the Silma, Silyb, and Mirel 1 varieties, silychristin was the most abundant flavonolignan, with concentrations of 5.98, 5.42 and 4.79 mg.g^−1^ DW, respectively, followed by silybin B and silybin A. Conversely, Silybin B predominated in the Moravia 55 (4.39 mg.g^−1^ DW) and Mirel 2 (5.22 mg.g^−1^ DW) varieties, with lower levels of silychristin and silybin A. The content of isosilybin A was two to four times higher than that of isosilybin B across all varieties. The most notable differences among the varieties were observed in silydianin levels, which ranged from 0.18 mg.g^−1^ DW (Mirel 2) to 3.01 mg.g^−1^ DW (Mirel 1). Moreover, it was not detected in the Moravia 55 variety. The HPLC-DAD chromatograms of the silymarin standard and the individual milk thistle varieties are shown in the Supplementary Material S1 (Figs. S1-S6).

**Table 1:** The content of silymarin complex components in milk thistle achenes of various varieties

Variety	Silychristin	Silydianin	Silybin A	Silybin B	Isosilybin A	Isosilybin B	Silymarin complex
	[mg.g^−1^ DW]						
Silma	5.98 ± 0.02 e	1.14 ± 0.02 c	4.61 ± 0.04 e	5.78 ± 0.04 c	2.07 ± 0.02 e	0.70 ± 0.01 c	20.28 ± 0.13 e
Silyb	5.42 ± 0.07 e	1.43 ± 0.02 d	4.15 ± 0.04 d	5.25 ± 0.05 b	1.93 ± 0.02 d	0.68 ± 0.01 c	18.86 ± 0.21 d
Mirel 1	4.79 ± 0.08 c	3.01 ± 0.004 e	3.44 ± 0.05 b	4.37 ± 0.08 a	1.87 ± 0.04 c	0.80 ± 0.02 d	18.27 ± 0.27 c
Mirel 2	4.38 ± 0.04 b	0.18 ± 0.03 b	3.61 ± 0.01 c	5.22 ± 0.03 b	1.48 ± 0.01 b	0.43 ± 0.01 b	15.31 ± 0.13 b
Moravia 55	3.57 ± 0.07 a	n.d.	3.11 ± 0.03 a	4.39 ± 0.03 a	1.26 ± 0.01 a	0.36 ± 0.01 a	12.69 ± 0.13 a

Note: Data are expressed as means ± standard deviation (*n* = 3). The silymarin complex was calculated as the sum of individual silymarin complex components. Means followed by the same letter are not statistically different at *p* ≤ 0.05 according to Fisher’s LSD test. n.d. – not detected

There is a general lack of studies on the flavonolignan composition of these specified milk thistle genotypes or from the same growing locations [13–15]. However, the few available studies have observed a similar trend in the distribution of major flavonolignans, typically with silybin B, silychristin, and silybin A as the predominant compounds. These studies generally report higher concentrations compared to our findings. The differences in flavonolignan levels may reflect both environmental influences and methodological variations, especially in extraction procedures. In our previous work [13], silychristin content in the Mirel variety ranged from 6.77 to 9.98 mg.g^−1^ DW depending on the field setup, followed by silybin B (6.24–8.88 mg.g^−1^ DW) and silybin A (5.30–7.68 mg.g^−1^ DW), with silydianin present at the lowest levels (0.13–0.73 mg.g^−1^ DW). Vágnerová et al. [14] found silybin B (13.51 mg.g^−1^) to be the dominant flavonolignan in variety Mirel, followed by silychristin (9.93 mg.g^−1^), silybin A (9.06 mg.g^−1^), and silydianin (3.21 mg.g^−1^) with isosilybin B (0.69 mg.g^−1^) as the least prevalent. A similar trend was also observed for the Silyb variety, where silybin B (14.19 mg.g^−1^) predominated, followed by silychristin (10.44 mg.g^−1^), and silybin A (9.66 mg.g^−1^). Silydianin was detected at an average concentration of 2.80 mg.g^−1^, and isosilybin B (0.72 mg.g^−1^) was represented at the lowest level. Silychristin (3.18–4.03 mg.g^−1^), silybin A (2.96–3.06 mg.g^−1^), and silybin B (5.92–7.04 mg.g^−1^) were also determined as the main flavonolignans of milk thistle achenes purchased in Slovakia by Habán et al. [15], with silydianin as the least abundant (0.91–1.76 mg.g^−1^). The milk thistle genotype was not specified. The silymarin complex composition of the Moravia 55 variety has not been yet reported. Variation in the composition of silymarin complex components in the milk thistle samples obtained from different countries during three years of cultivation was reported by Tran et al. [16]. While silydianin (0.69–9.16 mg.g^−1^ DW) and isosilybin A (1.85–3.27 mg.g^−1^ DW) were the two most abundant compounds in samples from Canada, Moldova, and Germany, silybin B (1.60–20.50 mg.g^−1^ DW) was predominant in samples from South and North Korea, followed by silychristin (0.73–13.73 mg.g^−1^ DW) and silybin A (0.45–10.42 mg.g^−1^ DW). The genotype of milk thistle samples was not specified. Bártová et al. [11] recently described the silymarin complex composition of the Tevasil and Tevadian cultivars. Silychristin, silybin A, silybin B, and isosilybin A were predominant in the achenes of the Tevasil variety, with average values of 24, 24, 36 and 10% of silymarin complex conent, respectively. Silydianin was the predominant component of the Tevadian variety, accounting for 62% of the silymarin complexcontent.

**Fig. 1:**
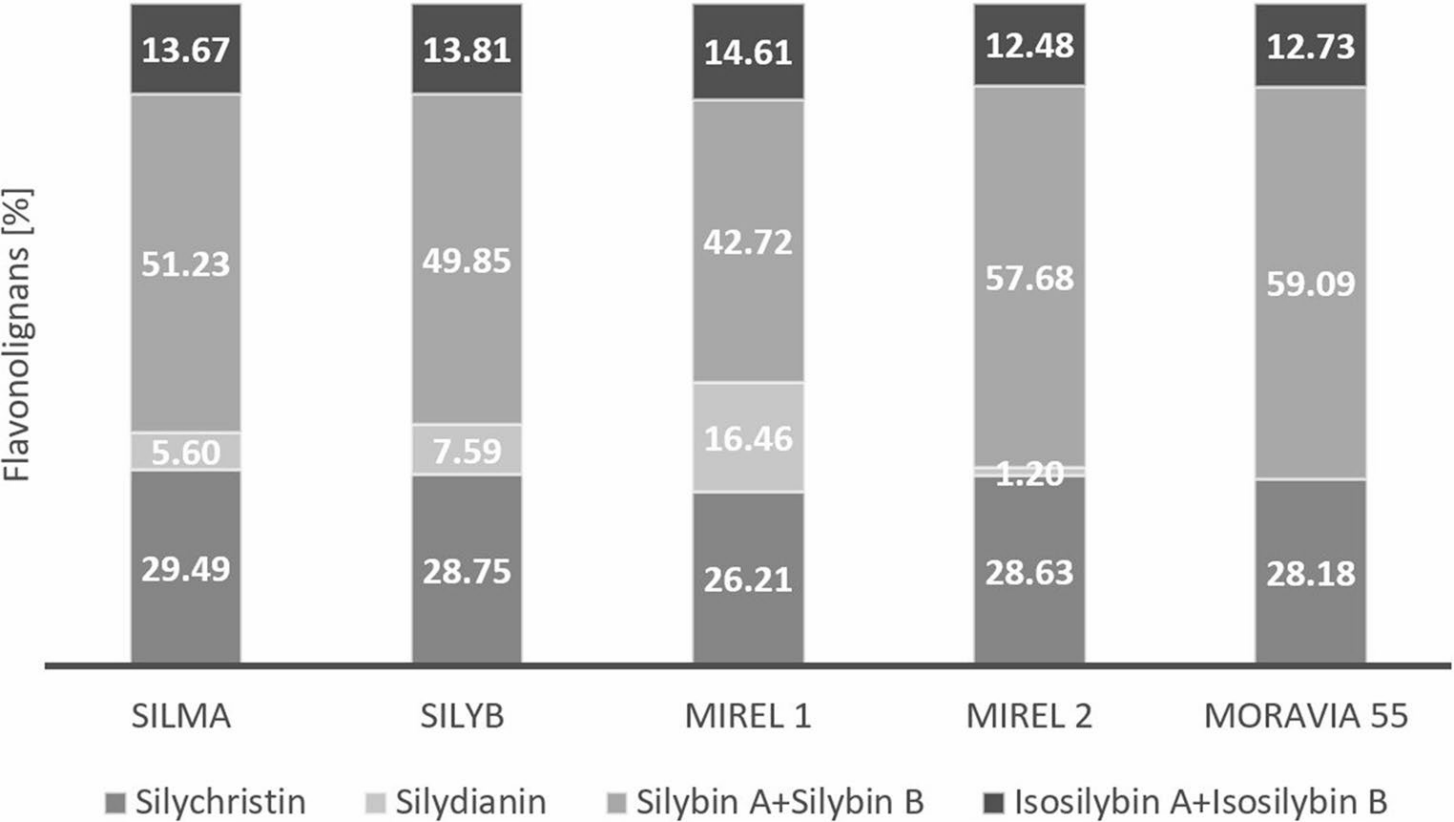
Mean flavonolignan content of milk thistle varieties expressed as a percentage of total silymarin complex content (*n* = 3)

The composition of flavonolignans within the silymarin complex is genetically determined. According to the literature, two chemotypes of *Silybum marianum* are recognized: chemotype A, characterized by a predominance of silybin and silychristin, and chemotype B, where silydianin is the dominant component [17]. The milk thistle varieties analyzed in this study can be classified as chemotype A since the content of silychristin and silybin (silybin A plus B) represents from 26.21 to 29.49% ad 42.72 to 59.09% o the total silymarin content, respectively (Fig. 1). As previously described, the differences in silymarin content between samples could be due to different genotypes, climatic and growing conditions, environmental factors (*e.g*., stress, sun exposure), as well as the time of harvest since the maturity of the seed has a significant effect on the composition of the silymarin complex [18–21].

### Oil Content and Fatty Acids Profile of Milk Thistle Achenes

**Fig. 2:**
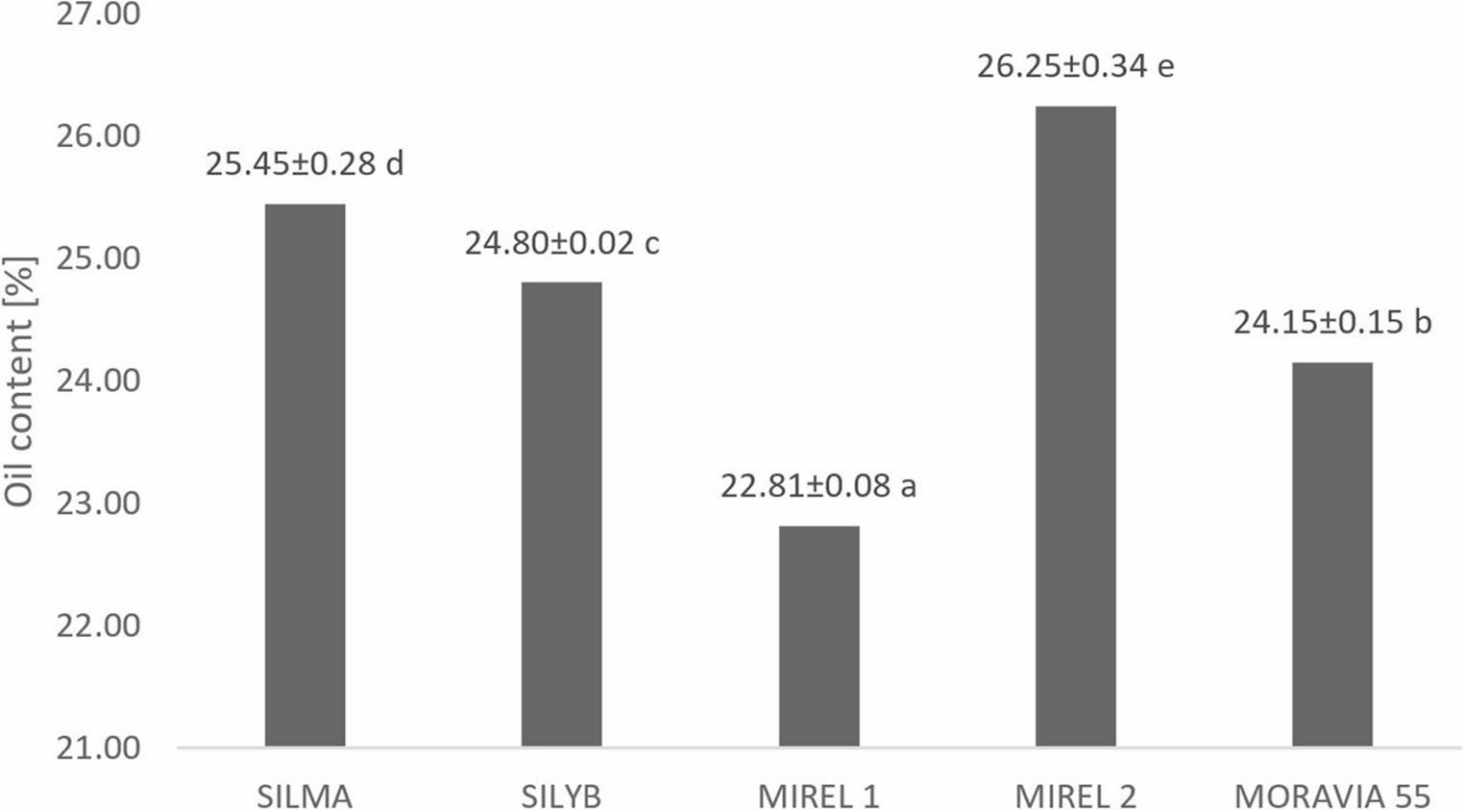
Oil yield of milk thistle achenes of various varieties. *Note: Data are expressed as means ± standard deviation (n = 3). Means followed by the same letter are not statistically different at p ≤ 0.05 according to Fisher’s LSD test*

The oil content and its fatty acid composition are key factors in determining the overall nutritional quality of the milk thistle oils. The oil content in milk thistle achenes varied from 22.81 to 26.25% depending on the variety with significant difference between them (*p* ˂ 0.001) (Fig. 2). The highest oil yield was observed for Mirel 2 variety, 26.25%, followed by Silma, 25.45%, Silyb, 24.80%, Moravia 55, 24.15%, and Mirel 1, 22.81%, varieties.

These oil contents are lower than those published in the previous study by Habán et al. [22] for the varieties Mirel, Silyb, and Silma (27.45–29.52%).The high oil content was found in the wild milk thistle seeds collected in Tunisia, at 30.50% [23] and 31.83% [24]. Theresults of this study are in good accordance with those of Sadowska et al. [25], who compared the effect of organic and conventional farming on the oil content of milk thistle, the percentage content in the varieties Mirel, Silma, and Silyb varied in the range of 22.94–23.25%, 23.54–24.96% and 22.60–23.9% respectively. Růžičková et al. [26] found the average oil content of Mirel and Silyb varieties to be 21%. These differencesmay be explained by previous findings that the seed oil content of milk thistle is influenced by geographical and climatic conditions, as well as by the maturity and genotype of the milk thistle [18, 26, 27]. The oil content of milk thistle is considerably lower than that reported for the major oilseeds such as sunflower (48–53%), sesame (48–55%), and rapesed (35–50%), but s comparable to that oflinseed (22–27%) or soybean (8–28%) [28].

The fatty acid composition of the individual oil variety is shown in Table 2, and GC-FID chromatograms are presented in the Supplementary Material S1 (Figs. S7-S12). The oils exhibited a high percentage composition of unsaturated fatty acids (UFA) with significant differences between varieties (*p* ˂ 0.001). The highest UFA composition was observed in Moravia 55 variety seed oil (83.80%), followed by Mirel 2 (82.38%), Mirel 1, Silyb, both (79.33%), and Silma (79.30%) variety seed oil. The percentage composition of polyunsaturated fatty acids (PUFA), which ranged from 53.76% (Silma) to 62.48% (Moravia 55), was higher than that of monounsaturated fatty acids (MUFA), varying from 21.32% (Moravia 55) to 25.54% (Silma). Significant differences (*p* ˂ 0.001) were also observed for the percentage composition of saturated fatty acids (SAFA) in the individual variety oils, ranging from 16.20% (Moravia 55) to 20.70% (Silma). Significant differences (*p* ˂ 0.001) were also observed for the PUFA/SAFA ratio, which varied from 2.60 (Silma) to 3.86 (Moravia 55). This information is important from a nutritional perspective, given the role of unsaturated fatty acids in cardiovascular disease prevention and their influence on various metabolic pathways [6, 7]. The results obtained are consistent with those of Růžičková et al. [26], who described the content of SAFA, MUFA, and PUFA of the Mirel and Silyb varieties in the range of 15.45–19.82%, 17.88–4.37%, and 5615 − 66.68%, respecively. Several other studies have reported similar results [23, 29, 30].

**Table 2:** Fatty acid profile of milk thistle seed oils of various varieties

Fatty acid	RT [min]	Variety				
		Silma	Silyb	Mirel 1	Mirel 2	Moravia 55
		[rel. %]				
Myristic acid (C14:0)	14.3	0.10 ± 0.002 ab	0.09 ± 0.003 a	0.09 ± 0.004 a	0.13 ± 0.007 c	0.11 ± 0.001 b
Myristoleic acid (C14:1)	14.6	n.d.	n.d.	n.d.	0.08 ± 0.007 b	0.08 ± 0.007 b
Pentadecenoic acid (C15:0)	15.2	0.04 ± 0.001 b	0.04 ± 0.004 b	0.04 ± 0.003 b	0.03 ± 0.001 ab	0.03 ± 0.001 a
*cis*−10-Pentadecenoic acid (C15:1)	15.6	0.06 ± 0.004 a	0.07 ± 0.005 a	0.06 ± 0.0001 a	0.15 ± 0.03 b	0.15 ± 0.02 b
Palmitic acid (C16:0)	16.2	9.06 ± 0.10 c	9.34 ± 0.03 d	9.32 ± 0.06 d	8.23 ± 0.04 b	7.23 ± 0.12 a
Palmitoleic acid (C16:1)	16.6	0.07 ± 0.001 a	0.08 ± 0.01 b	0.08 ± 0.002 ab	0.09 ± 0.004 c	0.07 ± 0.001 a
Stearic acid (C18:0)	18.8	5.70 ± 0.08 b	5.49 ± 0.06 b	5.64 ± 0.01 b	4.42 ± 0.16 a	4.27 ± 0.06 a
Oleic acid (C18:1n9c)	19.2	24.57 ± 0.11 d	23.85 ± 0.06 c	24.08 ± 0.001 c	20.81 ± 0.40 b	20.17 ± 0.08 a
Linoleic acid (C18:2n6c), ω−6	20.0	53.53 ± 0.22 a	54.25 ± 0.20 a	54.05 ± 0.12 a	60.21 ± 0.92 b	62.27 ± 0.31 c
α-Linolenic acid (18:3n3), ω−3	21.1	0.22 ± 0.01 bc	0.24 ± 0.01 c	0.23 ± 0.006 c	0.19 ± 0.01 a	0.21 ± 0.002 ab
Arachidic acid (C20:0)	22.2	3.10 ± 0.05 b	3.00 ± 0.05 b	2.99 ± 0.004 b	2.52 ± 0.17 a	2.34 ± 0.03 a
*cis*−11-Eicosenoic acid (C20:1)	22.7	0.84 ± 0.01 ab	0.85 ± 0.01 ab	0.82 ± 0.004 a	0.86 ± 0.02 b	0.85 ± 0.01 ab
Behenic acid (C22:0)	26.3	2.12 ± 0.07 b	2.10 ± 0.05 b	2.03 ± 0.02 b	1.80 ± 0.16 a	1.74 ± 0.02 a
Lignoceric acid (C24:0)	30.5	0.59 ± 0.01 bc	0.61 ± 0.02 c	0.56 ± 0.01 b	0.48 ± 0.02 a	0.48 ± 0.02 a
SAFA ∑		16.20 ± 0.25 a	20.70 ± 0.11 c	20.67 ± 0.14 c	20.67 ± 0.11 c	17.62 ± 0.55 b
MUFA ∑		21.32 ± 0.06 a	25.54 ± 0.12 d	24.84 ± 0.06 c	25.04 ± 0.01 c	21.98 ± 0.38 b
PUFA ∑		62.48 ± 0.31 c	53.76 ± 0.23 a	54.49 ± 0.20 a	54.29 ± 0.11 a	60.40 ± 0.93 b
UFA ∑		83.80 ± 0.37 c	79.30 ± 0.35 a	79.33 ± 0.26 a	79.33 ± 0.12 a	82.38 ± 1.31 b
PUFA/SAFA^*^		3.86 ± 0.08 d	2.60 ± 0.02 a	2.64 ± 0.03 b	2.63 ± 0.02 b	3.43 ± 0.16 c
AI^*^		0.09 ± 0.002 a	0.12 ± 0.001 c	0.12 ± 0.004 c	0.12 ± 0.001 c	0.11 ± 0.001 b
TI^*^		0.28 ± 0.01 a	0.37 ± 0.003 c	0.38 ± 0.001 c	0.38 ± 002 c	0.31 ± 0.01 b

Note: Data are expressed as means ± standard deviation (*n* = 3). Means followed by the same letter are not statistically different at *p* ≤ 0.05 according to Fisher’s LSD test

*n.d.* not detected, *RT *retention time. The identification of the compounds was based on a comparison of their retention times with those of the authentic standards. The position of the double bonds was not confirmed, *AI* Atherogenic Index, *TI* Thrombogenic Index

*dimensionless value

Regarding the individual fatty acid composition of the oils, statistically significant differences (*p* ˂ 0.05) were found for fatty acids depending on the milk thistle varieties analyzed.

The predominant fatty acids in the oils were linoleic (ω−6) and oleic acids, ranging from 53.53 to 62.27% and 20.17–24.57%, respectively. These were followed by palmitic acid (7.23–9.06%), stearic acid (4.27–5.70%), arachidic acid (2.34–3.10%), and behenic acid (1.74–2.12%). The percentage composition of myristic, myristoleic, pentadecanoic, cis-10-pentadecenoic, palmitoleic, α-linolenic (ω−3), cis-11-eicosenoic, and lignoceric acids was below 1%. The highest content of linoleic acid was noticed for the Moravia 55 variety, while the highest level of oleic acid was observed in the Silma variety. Myristoleic acid was detected only in the seed oil of varieties Mirel 2 and Moravia 55.

Similar results were reported by Růžičková et al. [26]. Linoleic acid ranged from 55.91 to 66.40%, leic acid from 16.26 to 22.89%, nd palmitic acid from 7.25 to 8.48%. he highest level of linoleic acid was found in the Mirel variety, and the highest oleic acid content was in the Silyb variety, as well as palmitic acid. As there is a lack of literature determining the fatty acid profile of individual milk thistle genotypes, numerous papers have characterized its profile in the oils of wild or unspecified milk thistle genotypes [29, 31–34]. In the study of Maalou et al. [29], the linoleic acid levels varied between 37.37 and 54.72%, whie the oleicacid content ranged from 13.28% to 24.79%. Addiionally, te palmitic acid content ranged from 9.12 to 13.09%. Zarrouk et al. [33] reported the fatty acid content in the oil of milk thistle collected from different regions of Tunisia, with linoleic acid ranging from 48.70 to 58.47%, oleic acd from 15.8 to 21.39%, and palmtic acid fom 6.25 to 13.06%. A high cntent of oeic acid, ranging from 28.54 to 35.85%, was foun in milk tistle achenes collected from various regions in Iran [34], with linoleic and palmitic acids ranging between 43.57 and 54.71% and 7.99–9.26%, respectvely. The diffeences between these results and those previously reported can be attributed to variations in geographical, climatic, and growing conditions, as well as to differences in the genotype and maturity of the milk thistle. A targeted investigation into the specific physiological and metabolic mechanisms and how these environmental factors influence lipid biosynthesis in specified milk thistle genotypes would be a valuable direction for future research.

The health-promoting potential of the fatty acid profiles of individual milk thistle varieties was further elucidated by calculating the Atherogenic index (AI) and Thrombogenic index (TI). These indexes reflect the balance between fatty acids that contribute to atherogenesis and thrombosis (saturated fatty acids) and those that offer protection against these factors (unsaturated fatty acids), with lower values indicating a greater potential to prevent cardiovascular disease and the formation of clots in the blood vessels. Significant differences were observed between varieties, with Moravia 55 exhibiting the most favorable AI and TI values (0.09 and 0.28, respectively), followed by Mirel 2 (0.11 and 0.31, respectively). The low AI and TI values in Moravia 55 and Mirel 2 are a direct consequence of their superior lipid composition, characterized by lower concentrations of SAFA and higher levels of MUFA and PUFA, especially oleic and linoleic acids. The AI and TI values of individual milk thistle varieties are comparable to those reported for commonly consumed vegetable oils, such as sunflower oil (AI: 0.06–0.07; TI: 0.18–0.28), rapeseed oil (AI: 0.05, TI: 0.09), olive oil (AI: 0.14–0.16, TI: 0.32–0.39), sesame oil (AI: 0.11–0.12, TI: 0.34–0.37), and linseed oil (AI: 0.06, TI: 0.05) [35, 36].

These findings align with the known breeding objectives of the individual cultivars. While the Silma and Silyb varieties are primarily grown for their high silymarin complex content, the Mirel variety is valued for its high oil content and specific fatty acid composition, as reflected by the results of this study. Moreover, this study provides valuable new insight into the Moravia 55 variety since it has not been characterized in the scientific literature.

### Multivariate Analysis of Milk Thistle Genotypes

**Fig. 3:**
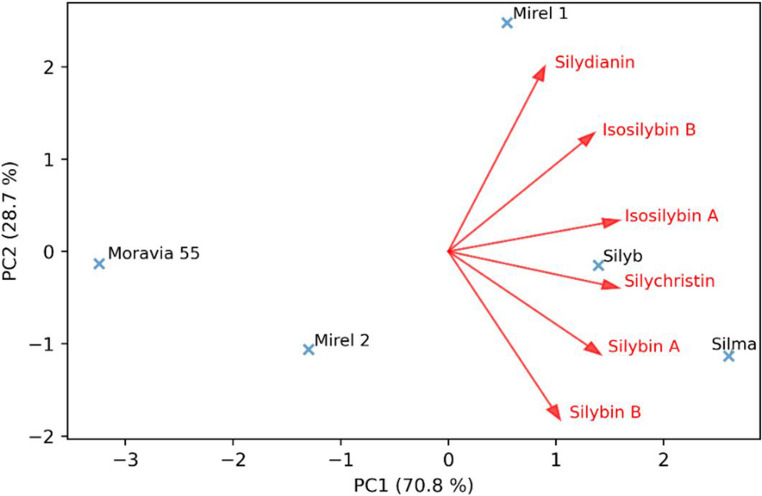
PCA biplot of silymarin compound distribution in milk thistle cultivars

**Fig. 4:**
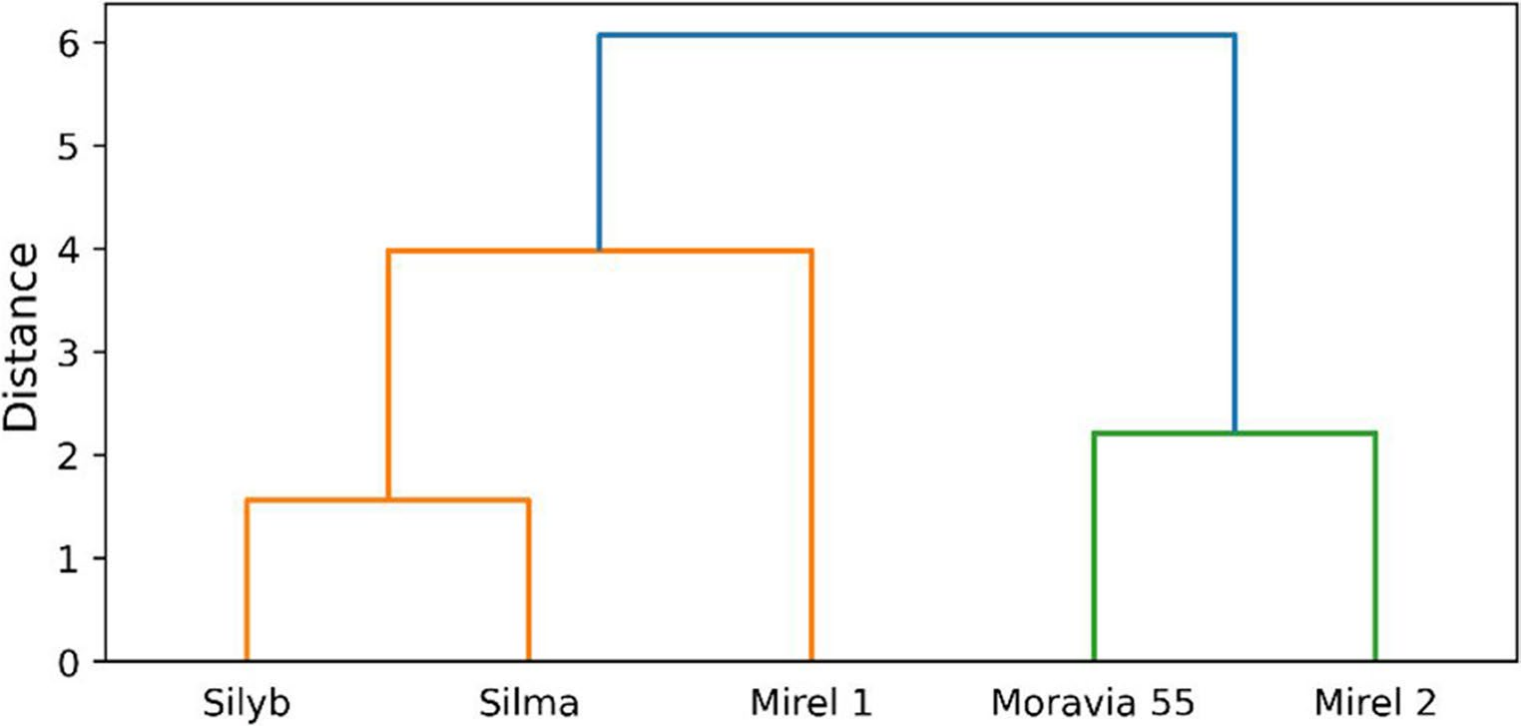
Cluster dendrogram of silymarin complex composition across milk thistle cultivars

The distribution characteristics of individual milk thistle components across genotypes were supported by the results of principal component analysis (PCA) and hierarchical cluster analysis. PCA biplot presenting the distribution of individual silymarin complex compounds across different milk thistle genotypes is shown in Fig. 3. The first two principal components together explain more than 95% of the total variability, with PC1 being the dominant axis (70.8%). Clear differences in silymarin composition are observed among the samples. Mirel 1 is strongly associated with higher levels of silydianin, isosilybin A, and isosilybin B, while Silma and Silyb are positively correlated with silybin A, silybin B, and silychristin. In contrast, Moravia 55 and Mirel 2 are located on the negative side of PC1, indicating a lower overall silymarin content in these samples. Moreover, the position of Moravia 55 along the negative axis of PC1 reflects its notably different distribution of silymarin components compared to the other genotypes, with minimal correlation to any specific silymarin compound. The dendrogram (Fig. 4) supports these trends, grouping Silyb, Silma, and Mirel 1, while Moravia 55 and Mirel 2 form a separate cluster, confirming the compositional diversity among the samples.

**Fig. 5:**
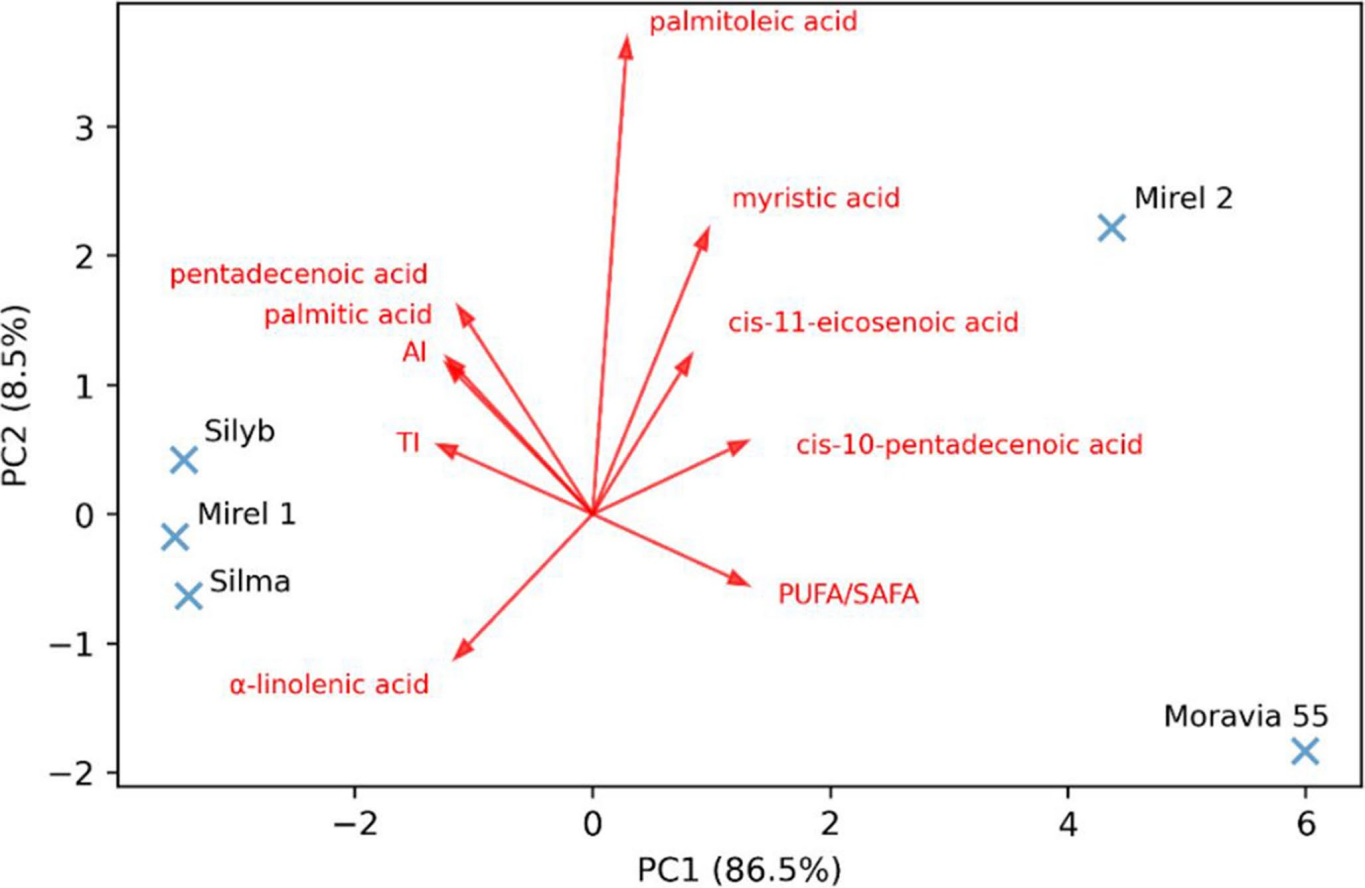
PCA biplot of fatty acid composition and nutritional indexes in seed oil samples of milk thistle cultivars

**Fig. 6:**
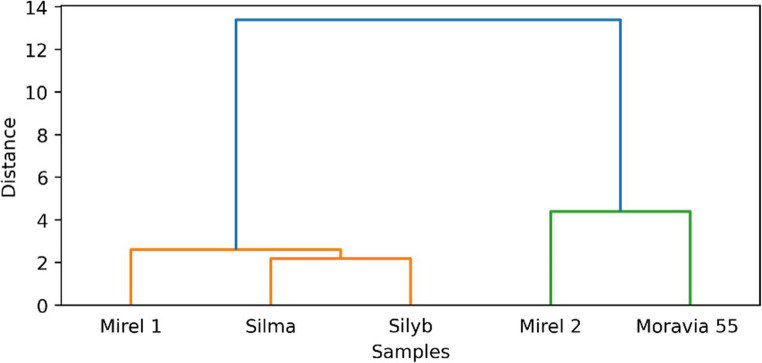
Cluster dendrogram of fatty acids composition and nutritional indexes across milk thistle cultivars

In addition, Fig. 5 illustrates the distribution of individual milk thistle oil samples based on their fatty acid profiles and selected nutritional indexes (PUFA/SAFA ratio, AI, and TI indexes). The first two principal components account for 95% of the total variance, with PC1 accounting for 86.5%. Sample Moravia 55 is clearly separated along the PC1 axis and shows a strong positive association with *cis*−10-pentadecenoic acid, PUFA/SAFA ratio, and cis-11-eicosenoic acid, indicating a unique fatty acid profile with potentially beneficial nutritional qualities. Mirel 2, located in the upper quadrant, correlates with palmitoleic acid and myristic acid, suggesting a distinct lipid profile compared to other samples. In contrast, Silyb, Mirel 1, and Silma cluster together in the negative region of PC1, associated with α-linolenic acid, palmitic acid, pentadecenoic acid, and higher TI values, indicating a similarity in their fatty acid profiles. The dendrogram further illustrates the relationships among the seed oil samples (Fig. 6) and reveals two principal clusters. The first cluster groups, Silyb, Silma, and Mirel 1, indicate a high degree of similarity in their fatty acid profiles and nutritional indexes. This is consistent with their proximity in the PCA biplot. Within this cluster, Silma and Silyb show the smallest distance, suggesting remarkably similar compositional characteristics. The second cluster consists of Mirel 2 and Moravia 55, which are distant from the first group. This reflects the distinctive fatty acid composition observed for these samples in the PCA, particularly the unique position of Moravia 55 along the PC1 axis. This clear separation into these two clusters supports the differentiation of seed oil varieties based on their lipid profiles, confirming the trends indicated by the PCA analysis.

Overall, the results revealed significant variability in the nutritional and phytochemical composition of the milk thistle samples. This may have implications for breeding research and functional food applications.

## Conclusions

The results of this study provide essential insights into the phytochemical composition of milk thistle seeds of various varieties. The highest silymarin complex content was found in the Silyb and Silma varieties, underlining their potential for pharmaceutical applications, particularly in the development of hepatoprotective formulations. Conversely, the Mirel variety showed a high oil yield, making it particularly valuable for applications in the food industry, especially in the production of functional foods and dietary oils. In addition, varieties with a higher linoleic acid content may be particularly suitable for dietary applications due to their beneficial effects on cardiovascular health, while a higher oleic acid content may enhance oxidative stability, which is crucial for oil storage and food industry applications. These findings may contribute to the strategic selection of milk thistle genotypes for specific pharmaceutical or nutritional purposes, and may also support targeted cultivation practices or the optimization of growing conditions to enhance the content of specific phytochemical compounds.

## Supplementary Information

Below is the link to the electronic supplementary material.

Supplementary file 1 (DOCX 305 KB)

## Data Availability

No datasets were generated or analysed during the current study.
