# Supplementary material for: Silymarin and Fatty Acid Profiles of Milk Thistle (Silybum marianum L.) Genotypes
Source: Plant Foods Hum Nutr. 2025 Sep 15;80(4):158. doi: 10.1007/s11130-025-01400-0 (PMC12433920; doi:10.1007/s11130-025-01400-0)
Supplement: Supplementary file 1 — (DOCX 305 KB) [file 11130_2025_1400_MOESM1_ESM.docx]

**Silymarin and Fatty Acid Profiles of Milk Thistle (*Silybum marianum* L.) Genotypes**

Barbora Kudláčková^a^, Petr Misák^c^, Helena Pluháčková^b*^

Plant Foods for Human Nutrition

^a^Institute of Analytical Chemistry of the Czech Academy of Sciences, Veveří 967/97, 602 00 Brno, Czech Republic.

^b^Department of Crop Science, Breeding and Plant Medicine, Mendel University in Brno, Zemědělská 1, 613 00 Brno, Czech Republic

^c^Faculty of Civil Engineering, Brno University of Technology, Veveří 331/95, 602 00 Brno, Czech Republic

Corresponding author: Helena Pluháčková; email: [helena.pluhackova@mendelu.cz](mailto:helena.pluhackova@mendelu.cz); address: Mendel University in Brno, Zemědělská 1, 613 00 Brno

**Materials and Methods**

**Chemicals**

Acetonitrile, methanol, n-hexane, and formic acid, all HPLC or GC-grade, were purchased from Riedel-de Haën (Seelze, Germany). Isooctane, p. a., and natrium chloride, p. a., were purchased from Lach-Ner, s.r.o. (Neratovice, Czech Republic). Water was purified with a reverse osmosis system Ultra Clear UV (Barsbüttel, Germany).

Sodium methoxide and 37 Component FAME Mix were obtained from Merck KGaA (Darmstadt, Germany). Silydianin, silychristin, silybin A, B, and isosilybin A, B standards were purchased from Extrasynthese (Genay Cedex, France). Stock solution of silymarin complex (0.5 g.L^-1^ each) was prepared in methanol and stored in the fridge at 4 °C.

**Ultrasound-assisted extraction of silymarin complex**

Ultrasound-assisted extraction was performed in an Elmasonic S 30H ultrasonic bath (Elma Schmidbauer GmbH, Singen, Germany). First, a portion (0.2 g) of ground achenes was defatted with 10 ml of isooctane for 5 min. The isooctane was then removed, and the achenes were extracted with 15 mL of methanol for 15 min. The obtained extract was put into a clean vial, and the extraction process was repeated with a fresh portion of methanol. Finally, 2 mL of the extract was filtered through a syringe filter and stored in the fridge until chromatographic analysis. The extraction process of each sample was performed in triplicate.

**Sample preparation for oil extraction and fatty acid determination**

A portion (1.0 g) of the individual ground achenes mixed in ratio 1:2 (v/v) with inert material (glass beads (600-800 µm)) was extracted with n-hexane using *one* PSE extractor (Applied Separations, USA) under extraction conditions set as follows: 11 mL stainless steel extraction cell, pressure of 15 MPa, temperature of 100 °C and extraction time of 2 x 10 minutes. After the extraction, the sample was flushed with a solvent volume equal to 60 % of the extraction cell's volume and purged with nitrogen for 90 seconds. The oils were recovered under the steam of nitrogen, then dried to a constant weight in a vacuum oven at 90 °C for 2 h, weighed, and stored in the fridge until further analysis. The extraction process was repeated in triplicate. The oil yield was calculated using the following equation: Oil yield (%) = (M/m) × 100, where M is the weight in grams of extracted oil and m is the weight in grams of the sample.

An aliquot of 100 mg of the oils obtained was weighed into a vial and 1.5 mL of 0.25 M sodium methoxide solution in methanol was added [1]. The vial was shaken for 30 s and then heated in an oven at 65 °C for 10 min. After that, 1 mL of NaCl saturated solution and 2 mL of hexane were added, and the mixture was again intensively shaken for 60 s. The upper hexane layer, containing the FAMEs, was pipetted into a 2 mL vial and GC-FID analysis was performed.

The atherogenic and thrombogenic index was calculated according to Ulbricht and Southgate [2].

*AI = (C12:0 + 4 × C14:0 + C16:0) / (ΣMUFA + ΣPUFA n-6 + ΣPUFA n-3)*

*TI = (C14:0 + C16:0 + C18:0) / (0.5×ΣMUFA + 0.5×ΣPUFA n-6 + 3×ΣPUFA n-3 + Σ(n-3/n-6)*

**GC-FID analysis of FAMEs**

The GC-FID analysis of methyl esters of fatty acids (FAMEs) was performed using Thermo Trace GC Ultra (Thermo Fisher Scientific Inc., Waltham, USA) equipped with DB-fastFAME capillary column (30 m × 0.25 mm i.d., film thickness 0.25 μm; Agilent J&W Scientific, Folsom, USA) [3]. The temperature programming was applied as follows: 50 °C (1 min), then increased to 185 °C at a rate of 10 °C/min (1 min), then to 210 °C at a rate of 2 °C/min, and then to 235 at a rate of 5 °C (2 min). The sample (1 µL) dissolved in n-hexane was injected at on-column mode, and helium was used as carrier gas at a flow rate of 1 mL/min. Injector and detector were maintained at 150 and 250 °C, respectively.

The peaks were identified based on their retention times using authentic standard fatty acids methyl esters and method of standard addition. Each sample was run in duplicate. The relative amounts (%) of compounds were calculated based on the GC peak area.

**Silymarin complex analysis by HPLC-DAD**

The HPLC-DAD analysis of obtained extracts was performed under the conditions previously described by the authors [4]. The target compounds were identified by comparing their retention times with those of authentic standards and the standard addition method. The external standard method was used for quantitative analysis.

Calibration solutions with five concentration levels ranging from 0.5 to 100 µg.mL^-1^ were prepared by diluting a concentrated mixture of individual compounds (0.5 mg.mL^-1^) according to their expected concentration in the extract. Each of these solutions was injected triplicate into HPLC-DAD system. The calibration curves were constructed by linear regression of the peak-area ratio of the individual standard versus its concentration. Good linearity with a correlation coefficient of *r^2^* ˃ 0.999 for all compounds was achieved in the investigated range. The limit of detection (LOD, *s/n=3*) was in the range of 0.060 – 0.240 µg.mL^-1^ for all analyzed compounds. Limit of quantification (LOQ, *s/n=10*)) was in the range of 0.200 – 0.800 µg.mL^-1^ for all analyzed compounds. The repeatability of the method (characterized by a relative standard deviation (RSD, (%))) was RSD ˂ 1 % for all analyzed compounds.

**Statistical analysis**

Data were processed using the program Statistica version 14.0 (StatSoft, Prague, Czech Republic). Significant differences between the results were determined by analysis of variance (ANOVA) and pairwise comparisons were made using Fisher’s LSD test. Principal Component Analysis (PCA) and hierarchical cluster analysis (HCA) were performed in Python using the scikit-learn and SciPy libraries, and visualized using Matplotlib. All statistical tests were performed at a significant level of *p* ˂ 0.001, *p* ˂ 0.01, and *p* ˂ 0.05.

**Results and Discussion**

**Table S1** Thousand seed weight of milk thistle samples.

| Sample | TSW [g] |
| --- | --- |
| Silma | 28.6 |
| Silyb | 24.0 |
| Mirel 1 | 26.2 |
| Mirel 2 | 24.2 |
| Moravia 55 | 23.0 |


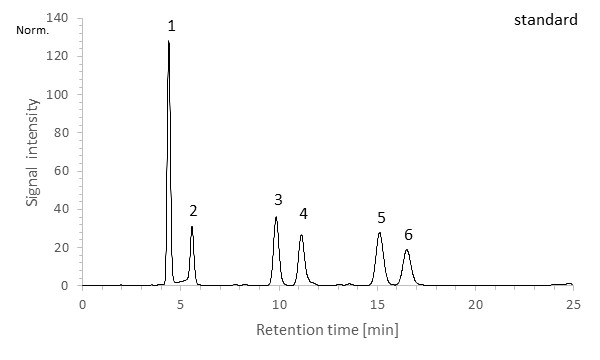


**Fig. S1** HPLC-DAD chromatogram of silymarin complex standard. Peak identification: 1) silychristin; 2) silydianin; 3) silybin A; 4) silybin B; 5) isosilybin A; 6) isosilybin B


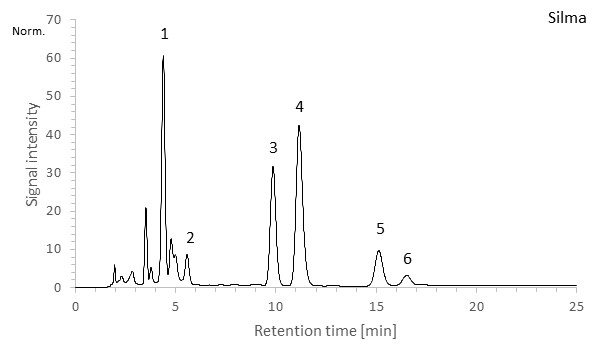


**Fig. S2** HPLC-DAD chromatogram of Silma variety. Peak identification: 1) silychristin; 2) silydianin; 3) silybin A; 4) silybin B; 5) isosilybin A; 6) isosilybin B


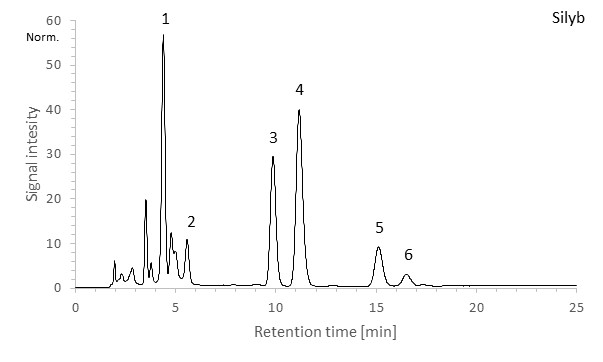


**Fig. S3** HPLC-DAD chromatogram of Silyb variety. Peak identification: 1) silychristin; 2) silydianin; 3) silybin A; 4) silybin B; 5) isosilybin A; 6) isosilybin B


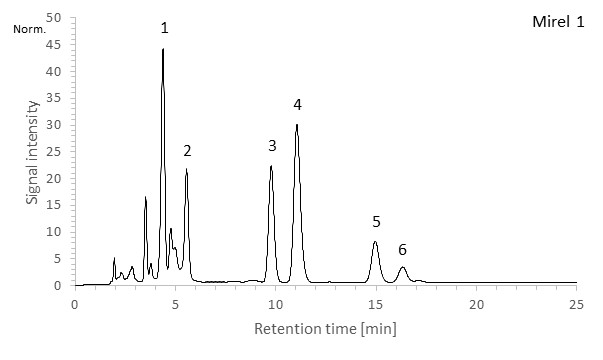


**Fig. S4** HPLC-DAD chromatogram of Mirel variety, sample 1. Peak identification: 1) silychristin; 2) silydianin; 3) silybin A; 4) silybin B; 5) isosilybin A; 6) isosilybin B


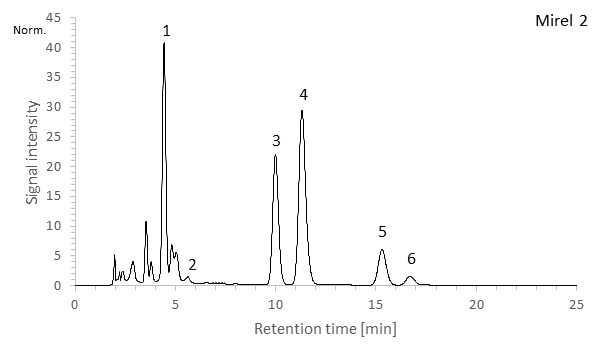


**Fig. S5** HPLC-DAD chromatogram of Mirel variety, sample 2. Peak identification: 1) silychristin; 2) silydianin; 3) silybin A; 4) silybin B; 5) isosilybin A; 6) isosilybin B


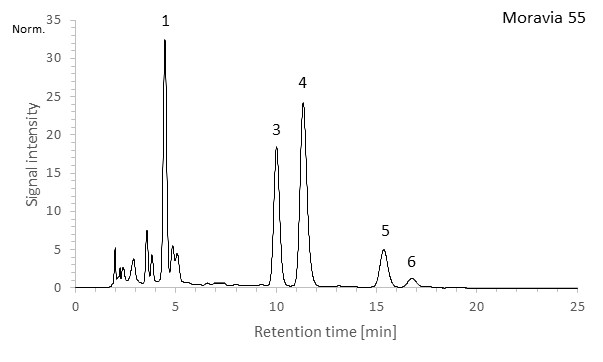


**Fig. S6** HPLC-DAD chromatogram of Moravia 55 variety. Peak identification: 1) silychristin; 2) silydianin; 3) silybin A; 4) silybin B; 5) isosilybin A; 6) isosilybin B


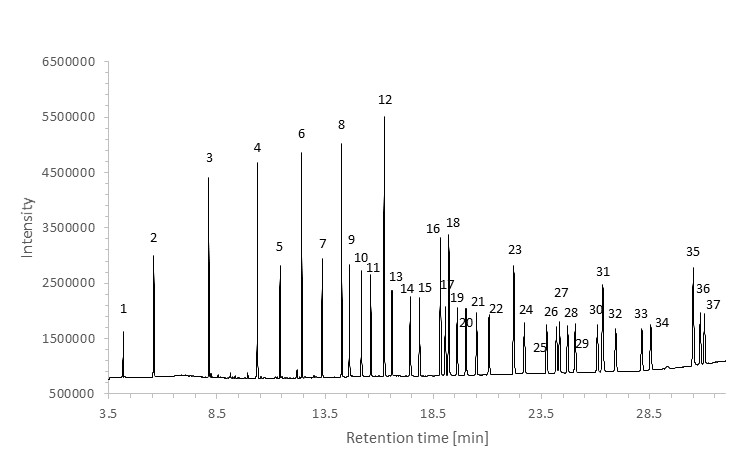


**Fig. S7** GC-FID chromatogram of FAMEs standard. Peak identification: 1) Butyric acid; 2) Caproic acid; 3) Caprylic acid; 4) Capric acid; 5) Undecanoic acid; 6) Lauric acid; 7) Tridecanoic acid; 8) Myristic acid; 9) Myristoleic acid; 10) Pentadecanoic acid; 11) *cis*-10-Pentadecenoic acid; 12) Palmitic acid; 13) Palmitoleic acid; 14) Heptadecanoic acid; 15) *cis*-10-Heptadecenoic acid; 16) Stearic acid; 17) Elaidic acid; 18) Oleic acid; 19) Linolelaidic acid; 20) Linoleic acid; 21) γ-Linolenic acid; 22) α-Linolenic acid; 23) Arachidic acid; 24) *cis*-11-Eicosenoic acid; 25) *cis*-11,14-Eicosadienoic acid; 26) Heneicosanoic acid; 27) *cis*-8,11,14-Eicosatrienoic acid; 28) Arachidonic acid; 29) *cis*-11,14,17-Eicosatrienoic acid; 30) *cis*-5,8,11,14,17-Eicosapentaenoic acid; 31) Behenic acid; 32) Erucic acid; 33) *cis*-13,16-Docosadienoic acid; 34) Tricosanoic acid; 35) Lignoceric acid; 36) Cervonic acid; 37) Nervonic acid.


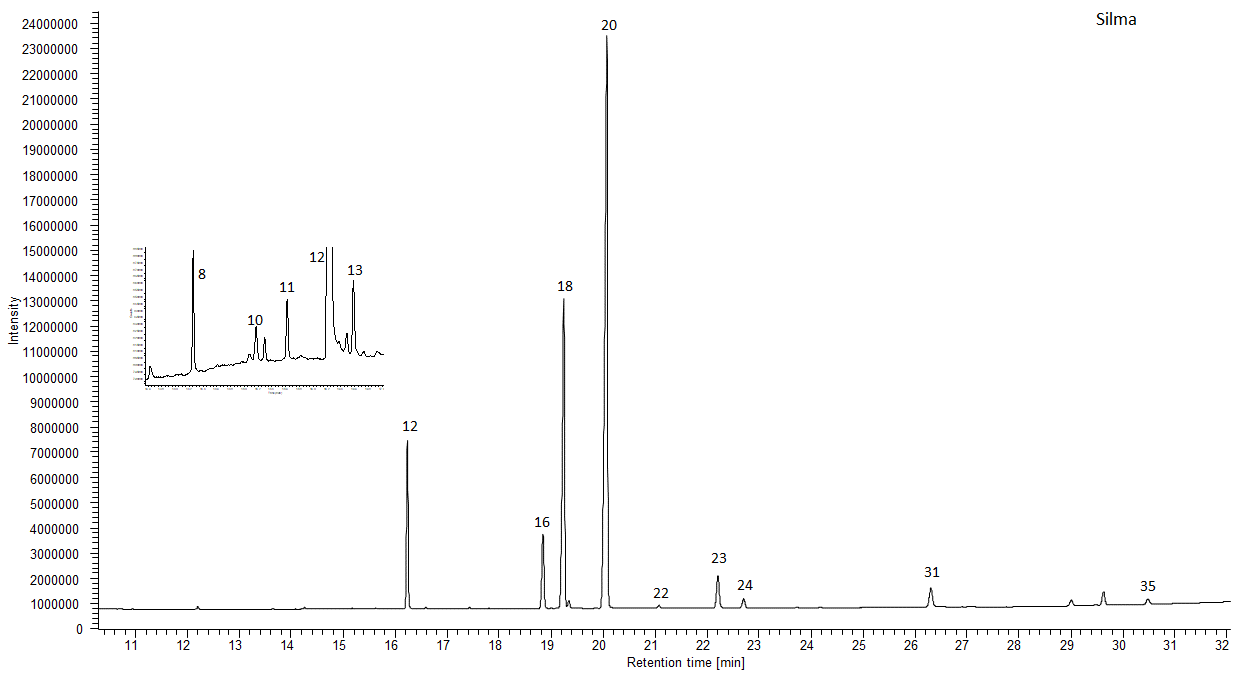


**Fig. S8** GC-FID chromatogram of Silma variety. Peak identification: 8) Myristic acid; 10) Pentadecanoic acid; 11) *cis*-10-Pentadecenoic acid; 12) Palmitic acid; 13) Palmitoleic acid; 16) Stearic acid; 18) Oleic acid; 20) Linoleic acid; 22) α-Linolenic acid; 23) Arachidic acid; 24) *cis*-11-Eicosenoic acid; 31) Behenic acid; 35) Lignoceric acid.


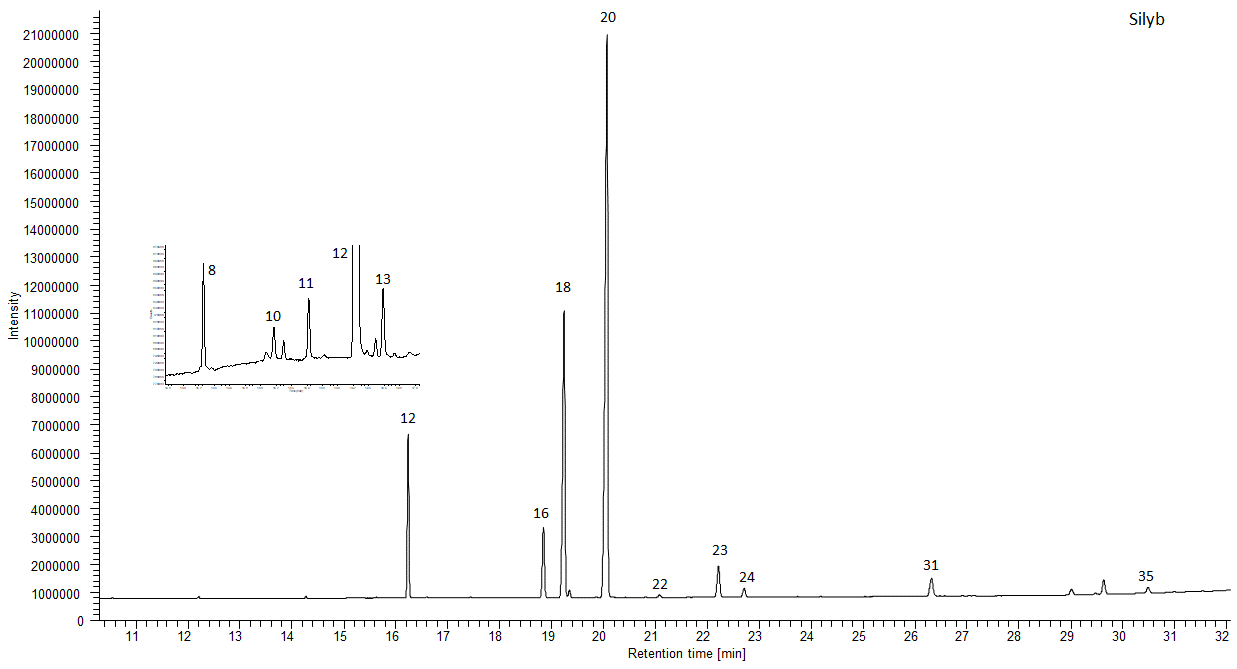


**Fig. S9** GC-FID chromatogram of Silyb variety. Peak identification: 8) Myristic acid; 10) Pentadecanoic acid; 11) *cis*-10-Pentadecenoic acid; 12) Palmitic acid; 13) Palmitoleic acid; 16) Stearic acid; 18) Oleic acid; 20) Linoleic acid; 22) α-Linolenic acid; 23) Arachidic acid; 24) *cis*-11-Eicosenoic acid; 31) Behenic acid; 35) Lignoceric acid.


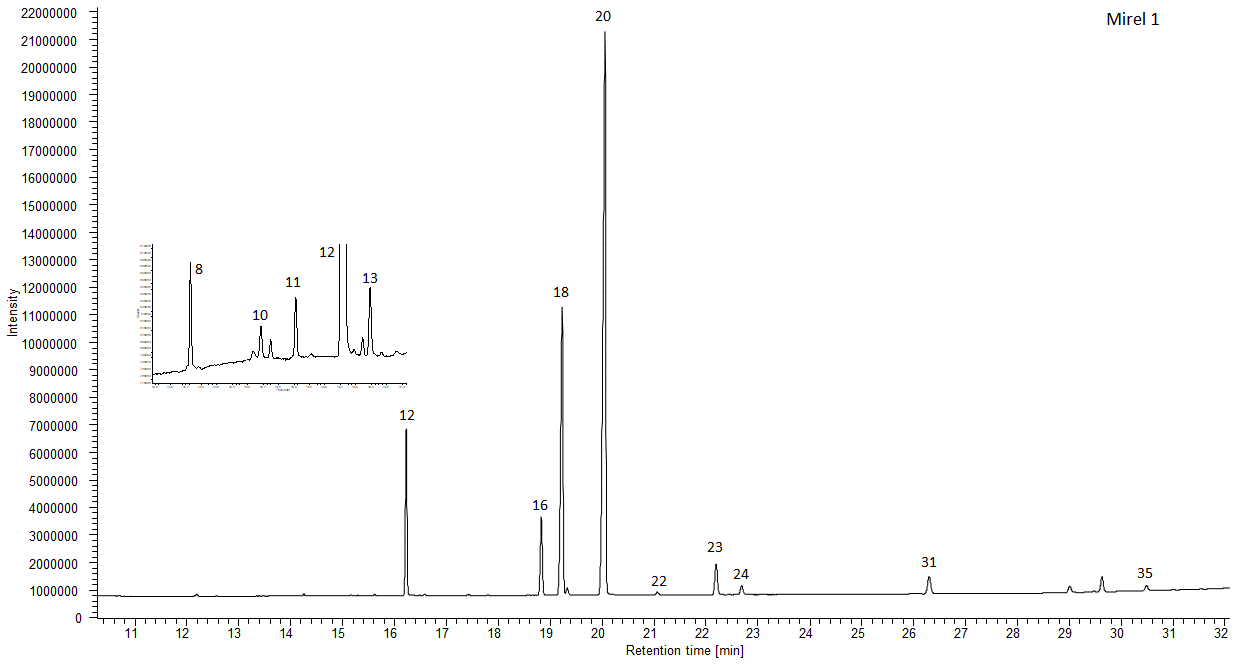


**Fig. S10** GC-FID chromatogram of Mirel variety, sample 1. Peak identification: 8) Myristic acid; 10) Pentadecanoic acid; 11) *cis*-10-Pentadecenoic acid; 12) Palmitic acid; 13) Palmitoleic acid; 16) Stearic acid; 18) Oleic acid; 20) Linoleic acid; 22) α-Linolenic acid; 23) Arachidic acid; 24) *cis*-11-Eicosenoic acid; 31) Behenic acid; 35) Lignoceric acid.


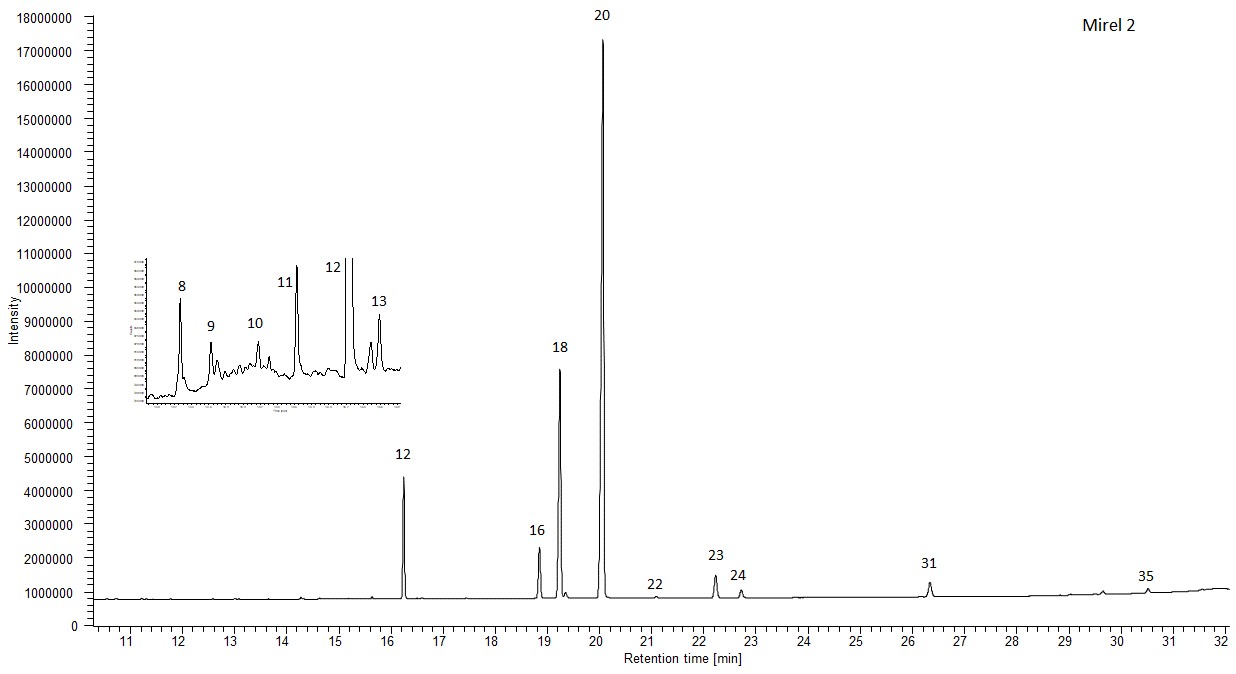


**Fig. S11** GC-FID chromatogram of Mirel variety, sample 2. Peak identification: 8) Myristic acid; 9) Myristoleic acid; 10) Pentadecanoic acid; 11) *cis*-10-Pentadecenoic acid; 12) Palmitic acid; 13) Palmitoleic acid; 16) Stearic acid; 18) Oleic acid; 20) Linoleic acid; 22) α-Linolenic acid; 23) Arachidic acid; 24) *cis*-11-Eicosenoic acid; 31) Behenic acid; 35) Lignoceric acid.


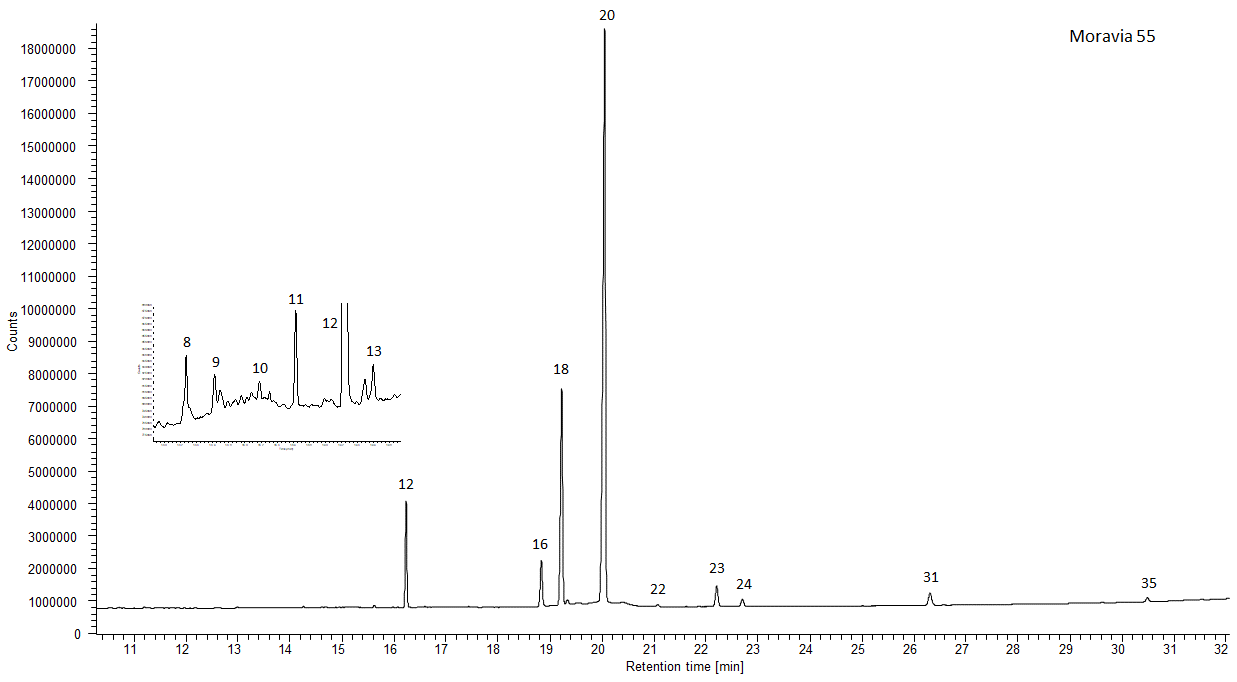


**Fig. S12** GC-FID chromatogram of Moravia 55 variety. Peak identification: 8) Myristic acid; 9) Myristoleic acid; 10) Pentadecanoic acid; 11) *cis*-10-Pentadecenoic acid; 12) Palmitic acid; 13) Palmitoleic acid; 16) Stearic acid; 18) Oleic acid; 20) Linoleic acid; 22) α-Linolenic acid; 23) Arachidic acid; 24) *cis*-11-Eicosenoic acid; 31) Behenic acid; 35) Lignoceric acid.

**References**

1. Galuszka J, Vostalova J, Cervena B, et al (2015) Omega‐3 fatty acid supplementation candidates can be selected using fatty acid profiling. Eur J Lipid Sci Technol 117:601–607. <https://doi.org/10.1002/ejlt.201400213>
2. Ulbricht TLV, Southgate DAT (1991) Coronary heart disease: seven dietary factors. The Lancet 338:985–992. <https://doi.org/10.1016/0140-6736(91)91846-M>
3. Zou Y, Wu H. Improving the analysis of 37 fatty acid methyl esters using three types of capillary GC columns. Agilent Technologies Application Note 5991-8706EN. Santa Clara, CA; 2023.
4. Pluháčková H, Kudláčková B, Svojanovská L, et al (2023) Effect of Field Trial on Silymarin Complex Composition and Antioxidant Assessment of Milk Thistle (*Silybum marianum* L. Gaertner). Plant Foods Hum Nutr 78:691–697. https://doi.org/10.1007/s11130-023-01101-6
